# Long-Term Risk of Incident Type 2 Diabetes and Measures of Overall and Regional Obesity: The EPIC-InterAct Case-Cohort Study

**DOI:** 10.1371/journal.pmed.1001230

**Published:** 2012-06-05

**Authors:** The InterAct Consortium

**Affiliations:** The George Institute for International Health, Australia

## Abstract

**Background:**

Waist circumference (WC) is a simple and reliable measure of fat distribution that may add to the prediction of type 2 diabetes (T2D), but previous studies have been too small to reliably quantify the relative and absolute risk of future diabetes by WC at different levels of body mass index (BMI).

**Methods and Findings:**

The prospective InterAct case-cohort study was conducted in 26 centres in eight European countries and consists of 12,403 incident T2D cases and a stratified subcohort of 16,154 individuals from a total cohort of 340,234 participants with 3.99 million person-years of follow-up. We used Prentice-weighted Cox regression and random effects meta-analysis methods to estimate hazard ratios for T2D. Kaplan-Meier estimates of the cumulative incidence of T2D were calculated. BMI and WC were each independently associated with T2D, with WC being a stronger risk factor in women than in men. Risk increased across groups defined by BMI and WC; compared to low normal weight individuals (BMI 18.5–22.4 kg/m^2^) with a low WC (<94/80 cm in men/women), the hazard ratio of T2D was 22.0 (95% confidence interval 14.3; 33.8) in men and 31.8 (25.2; 40.2) in women with grade 2 obesity (BMI≥35 kg/m^2^) and a high WC (>102/88 cm). Among the large group of overweight individuals, WC measurement was highly informative and facilitated the identification of a subgroup of overweight people with high WC whose 10-y T2D cumulative incidence (men, 70 per 1,000 person-years; women, 44 per 1,000 person-years) was comparable to that of the obese group (50–103 per 1,000 person-years in men and 28–74 per 1,000 person-years in women).

**Conclusions:**

WC is independently and strongly associated with T2D, particularly in women, and should be more widely measured for risk stratification. If targeted measurement is necessary for reasons of resource scarcity, measuring WC in overweight individuals may be an effective strategy, since it identifies a high-risk subgroup of individuals who could benefit from individualised preventive action.

*Please see later in the article for the Editors' Summary*

## Abstract 2

A collaborative re-analysis of data from the InterAct case-control study conducted by Claudia Langenberg and colleagues has established that waist circumference is associated with risk of type 2 diabetes, independently of body mass index.

## Editors' Summary

**Background:**

Worldwide, more than 350 million people have diabetes, and this number is increasing rapidly. Diabetes is characterized by dangerous levels of glucose (sugar) in the blood. Blood sugar levels are usually controlled by insulin, a hormone that the pancreas releases after meals (digestion of food produces glucose). In people with type 2 diabetes (the commonest form of diabetes), blood sugar control fails because the fat and muscle cells that normally respond to insulin by removing sugar from the blood become insulin resistant. Type 2 diabetes can be controlled with diet and exercise, and with drugs that help the pancreas make more insulin or that make cells more sensitive to insulin. The long-term complications of diabetes, which include an increased risk of heart disease and stroke, reduce the life expectancy of people with diabetes by about 10 years compared to people without diabetes.

**Why Was This Study Done?:**

A high body mass index (BMI, a measure of body fat calculated by dividing a person's weight in kilograms by their height in meters squared) is a strong predictor of type 2 diabetes. Although the risk of diabetes is greatest in obese people (who have a BMI of greater than 30 kg/m^2^), many of the people who develop diabetes are overweight—they have a BMI of 25–30 kg/m^2^. Healthy eating and exercise reduce the incidence of diabetes in high-risk individuals, but it is difficult and expensive to provide all overweight and obese people with individual lifestyle advice. Ideally, a way is needed to distinguish between people with high and low risk of developing diabetes at different levels of BMI. Waist circumference is a measure of fat distribution that has the potential to quantify diabetes risk among people with different BMIs because it estimates the amount of fat around the abdominal organs, which also predicts diabetes development. In this case-cohort study, the researchers use data from the InterAct study (which is investigating how genetics and lifestyle interact to affect diabetes risk) to estimate the long-term risk of type 2 diabetes associated with BMI and waist circumference. A case-cohort study measures exposure to potential risk factors in a group (cohort) of people and compares the occurrence of these risk factors in people who later develop the disease and in a randomly chosen subcohort.

**What Did the Researchers Do and Find?:**

The researchers estimated the association of BMI and waist circumference with type 2 diabetes from baseline measurements of the weight, height, and waist circumference of 12,403 people who subsequently developed type 2 diabetes and a subcohort of 16,154 participants enrolled in the European Prospective Investigation into Cancer and Nutrition (EPIC). Both risk factors were independently associated with type 2 diabetes risk, but waist circumference was a stronger risk factor in women than in men. Obese men (BMI greater than 35 kg/m^2^) with a high waist circumference (greater than 102 cm) were 22 times more likely to develop diabetes than men with a low normal weight (BMI 18.5–22.4 kg/m^2^) and a low waist circumference (less than 94 cm); obese women with a waist circumference of more than 88 cm were 31.8 times more likely to develop type 2 diabetes than women with a low normal weight and waist circumference (less than 80 cm). Importantly, among overweight people, waist circumference measurements identified a subgroup of overweight people (those with a high waist circumference) whose 10-year cumulative incidence of type 2 diabetes was similar to that of obese people.

**What Do These Findings Mean?:**

These findings indicate that, among people of European descent, waist circumference is independently and strongly associated with type 2 diabetes, particularly among women. Additional studies are needed to confirm this association in other ethnic groups. Targeted measurement of waist circumference in overweight individuals (who now account for a third of the US and UK adult population) could be an effective strategy for the prevention of diabetes because it would allow the identification of a high-risk subgroup of people who might benefit from individualized lifestyle advice.

**Additional Information:**

Please access these web sites via the online version of this summary at http://dx.doi.org/10.1371/journal.pmed.1001230.

- The US National Diabetes Information Clearinghouse provides information about diabetes for patients, health care professionals, and the general public, including detailed information on diabetes prevention (in English and Spanish)
- The US Centers for Disease Control and Prevention provides information on all aspects of overweight and obesity (including some information in Spanish)
- The UK National Health Service Choices website provides information for patients and carers about type 2 diabetes, about the prevention of type 2 diabetes, and about obesity; it also includes peoples stories about diabetes and about obesity
- The charity Diabetes UK also provides detailed information for patients and carers, including information on healthy lifestyles for people with diabetes, and has a further selection of stories from people with diabetes; the charity Healthtalkonline has interviews with people about their experiences of diabetes
- More information on the InterAct study is available
- MedlinePlus provides links to further resources and advice about diabetes and diabetes prevention and about obesity (in English and Spanish)

## Introduction

A higher body mass index (BMI) is a strong predictor of type 2 diabetes (T2D), with a linear increase in diabetes risk across the whole spectrum of BMI [1]. Although diabetes risk is highest in obese people with BMI≥30 kg/m^2^, a great proportion of future cases comes from the large population of overweight individuals with a BMI between 25 and 30 kg/m^2^ [2]. Recent national figures from the US and UK suggest that at least a third of the population is now overweight and another third (24% UK) is obese [3],[4], with severe implications for the future burden of diabetes.

The Diabetes Prevention Program has shown that individual-level lifestyle intervention can reduce the incidence of diabetes by over 50% in high-risk individuals [5], an effect that persists for at least 10 y [6]. Current clinical practice generally relies on measurement of BMI to identify individuals at increased risk of diabetes and other adiposity-related morbidity and mortality. However, due to their high prevalence, it is financially and logistically difficult to test and intervene on all overweight and obese individuals.

BMI provides no information about body fat distribution, which distinguishes the large amount of adipose tissue located subcutaneously from the smaller amount of intra-abdominal visceral fat, known to predict the development of diabetes over and above BMI [2],[7],[8]. Waist circumference (WC) is a simple measure that can be used to diagnose abdominal obesity and identify individuals at increased risk of T2D [9]. Information about WC is therefore likely to be useful in distinguishing high- and low-risk individuals at different levels of BMI, which is important for targeting those at highest absolute risk for individually focused lifestyle intervention to prevent T2D.

However, WC is not routinely assessed in clinical practice for a range of reasons [10]. Although measurement is relatively simple and cheap, it does require some training and standardisation, and this has been cited as one reason for its limited use. Another explanation is that practitioners do not appreciate the relevance of the additional information that is derived from measuring WC over and above BMI [10]. This may be because earlier studies were generally too small to estimate T2D incidence rates at different levels of BMI and WC with the precision required to guide clinical decision making or inform policy recommendations. In addition, men and women differ in the distribution of their overall and abdominal body fat, but only large-scale studies including men and women are adequately powered to investigate sex differences in associations of BMI and WC with T2D with confidence.

We use data from the European InterAct study, a case-cohort study of 12,403 cases of incident T2D and a subcohort of 16,154 participants, conducted in 26 centres in eight European countries to estimate the relative and cumulative risk of diabetes at different levels of BMI and WC, separately in men and women.

## Methods

### Population

The design and methods of the InterAct case-cohort study have previously been described in detail [11]. InterAct Consortium partners identified individuals with T2D in European Prospective Investigation into Cancer and Nutrition (EPIC) cohorts between 1991 and 2007 from eight of the ten countries participating in EPIC (26 centres). Prevalent diabetes was identified on the basis of baseline self-report of a history of diabetes, doctor-diagnosed diabetes, diabetes drug use, or evidence of diabetes after baseline with a date of diagnosis earlier than the baseline recruitment date. All ascertained cases with any evidence of diabetes at baseline were excluded. Ascertainment of incident T2D involved a review of the existing EPIC datasets at each centre using multiple sources of evidence including self-report, linkage to primary-care registers, secondary-care registers, medication use (drug registers), hospital admissions and mortality data. Information from any follow-up visit or external evidence with a date later than the baseline visit was used. To increase the specificity of the case definition, we sought further evidence for all cases with information on incident T2D from fewer than two independent sources at a minimum, which included individual medical records review in some centres. Cases in Denmark and Sweden were not ascertained by self-report, but identified via local and national diabetes and pharmaceutical registers, and hence all ascertained cases were considered to be verified. Follow-up was censored at the date of diagnosis, 31 December 2007, or the date of death, whichever occurred first. A total of 340,234 participants of European descent were followed up for 3.99 million person-years (mean [range] of follow-up 11.7 [0–17.5] y), during which 12,403 verified incident cases of T2D were identified [11]. Individuals without stored blood (*n* = 109,625) or without reported diabetes status (*n* = 5,821) were excluded. A centre-stratified, random subcohort of 16,835 individuals was selected; after exclusion of 548 individuals with prevalent diabetes and 133 with unknown diabetes status, the subcohort included 16,154 individuals for analysis. Due to the random selection, this subcohort also included a random set of 778 individuals who had developed incident T2D during follow-up. Participants in the random subcohort were similar to all EPIC participants eligible for inclusion in InterAct [11]. InterAct cases were followed-up for a mean (standard deviation [SD]) of 6.9 (3.3) y; 49.8% were men. The overall incidence in InterAct was 3.8 per 1,000 person-years of follow-up; country-specific rates are included in the InterAct cohort description [11].

### Measurements

Weight and height were measured with participants not wearing shoes and in light clothing or underwear in the majority of centres, as described previously [12]. WC was measured either at the narrowest circumference of the torso or at the midpoint between the lower ribs and the iliac crest. Hip circumference was measured horizontally at the level of the largest lateral extension of the hips or over the buttocks. For a subset of the Oxford (UK) participants (*n* = 363), only self-reported waist and hip circumferences were available. Each participant's body weight and waist and hip circumferences were corrected for the clothing worn during measurement in order to reduce heterogeneity due to protocol differences among centres [13]. Correction included adjustment for self-reporting in Oxford participants using a prediction equation based on a comparison of self-reported and measured data in a sample of 5,000 of the Oxford general population [12],[14]. BMI was calculated as weight (in kilograms)/height (in metres) squared. Waist-hip ratio was calculated and expressed as a percentage. Measures of waist or hip circumference were not performed at the centre in Umea, Sweden (*n* = 1,845), and were missing in an additional 173 and 193 InterAct participants for waist and hip, respectively.

As part of EPIC, standardised information on education and smoking status was collected by questionnaire at baseline [15]. Physical activity was assessed using a brief questionnaire covering occupation and recreational activity [16],[17].

### Statistical Analysis

Characteristics of the subcohort are described using summary statistics (means, SDs, frequencies, and percentages) separately for men and women. Associations between anthropometric variables and the hazard of diabetes were estimated using Prentice-weighted Cox regression models with age as the underlying time scale, separately within each centre and then combined across centres using random effects meta-analysis [11]. We calculated internally derived sex-specific standardised scores based on means and SDs within the subcohort for each anthropometric measure. We divided study participants into normal weight (BMI 18.5–24.9 kg/m^2^), overweight (25.0–29.9 kg/m^2^), or obese (≥30 kg/m^2^) based on current World Health Organization criteria [18], and used sex-specific cut-offs to define WC as normal (<94 cm [<34.6 inches] in men and <80 cm [31.5 inches] in women), moderately increased (94–102 cm [34.6–40 inches] in men and 80–88 cm [31.5–35 inches] in women), or large (≥102 cm [≥40 inches] in men and ≥88 cm [≥35 inches] in women) [18],[19]. We excluded 189 participants (172 subcohort members and 17 cases from outside the subcohort) who were underweight (BMI<18.5 kg/m^2^) from all analyses. Using standardised, continuous measures and categorical BMI and WC variables, we compared the effect estimates for associations between each anthropometric measure and the risk of diabetes, separately for men and women, before and after adjustments. Adjustments included other anthropometric measures, smoking, education, and physical activity, as specified for all models in the corresponding tables.

Where heterogeneity was observed in the effect estimates between centres, meta-regression was used to explore the extent to which the average age, BMI, or WC in each centre explained the heterogeneity. To assess whether the effect estimate of WC differed between men and women, a sex by WC (continuous variable) interaction term was included in the centre-specific Prentice-weighted Cox regression models, and the estimated interaction coefficients were then combined across centres using random effects meta-analysis. A similar analysis was performed to assess the evidence for a BMI group by WC group interaction using standard, clinical cut-offs.

To investigate the hazard ratio (HR) of diabetes by BMI and WC levels in more detail, we further subdivided study participants who had measures of BMI and WC into six BMI groups (18.5–22.4, 22.5–24.9, 25–27.4, 27.5–29.9, 30.0–34.9, and ≥35 kg/m^2^). Analyses were performed using Stata version 12 (StataCorp) Within Stata, the –st- suite of commands for performing survival analysis was used.

To estimate the cumulative incidence of diabetes we performed bootstrap sampling using the Stata bsample command to recreate the full cohort by resampling with replacement from the subcohort, according to the BMI and WC distributions within the subcohort. This made it possible to estimate absolute cumulative incidences (one minus the Kaplan-Meier estimate of the survivor function) for normal, increased, and large WC groups separately within the groups of normal, overweight, and obese men and women.

## Results

Characteristics of men and women who were part of the subcohort are shown in Tables 1 and 2. A total of 50.0% of men and 33.8% of women were overweight, and 16.4% of men and 15.8% of women were obese. Overweight or obese men and women were shorter and had larger WCs and waist-hip ratios than participants with normal baseline BMI (all *p*<0.001). Overweight or obese men and women were more likely to be physically inactive and to be educated at primary school level or less (all *p*<0.001). While obese men were less likely to be never smokers and more likely to be former smokers, the opposite was observed in obese women, who were more likely to be never smokers and less likely to be former or current smokers (all *p*<0.001).

**Table 1:** Characteristics of the subcohort by BMI group in men of the InterAct study.

Characteristic	Missing (Percent)	Normal, 18.5–24.9 kg/m^2^ (*n* = 2,026)		Overweight, 25.0–29.9 kg/m^2^ (*n* = 3,028)		Obese, ≥30.0 kg/m^2^ (*n* = 996)		Total (*n* = 6,050)	
**Age (years)**	0	52.0	9.9	53.2	8.5	53.6	7.9	52.9	8.9
**Weight (kilograms)**	0.2	71.3	7.2	82.0	7.7	96.0	11.0	80.7	11.7
**Height (centimetres)**	0.2	175.8	7.2	173.5	7.1	171.9	7.7	174.0	7.4
**WC (centimetres)** a	3.4	86.2	6.3	96.3	6.3	108.5	7.6	95.1	10.1
**Waist-hip ratio (percent)** a	3.4	90.2	5.4	94.9	5.5	99.0	5.8	94.1	6.4
**Weight at age 20 y (kilograms)**	22.0	67.3	7.6	70.4	8.2	75.1	10.0	69.8	8.6
**Change in weight (kilograms)**	22.0	4.5	6.8	13.3	7.8	24.2	10.5	11.4	10.2
**Duration of follow-up (years)**	0.0								
Non-case		12.1	2.3	12.2	2.2	12.1	2.5	12.2	2.3
Incident diabetes case		6.8	3.3	7.3	3.1	6.8	3.3	7.1	3.2
**WC groups** a	3.4								
<94 cm		77.7	1,574	30.6	927	1.1	11	41.7	2,529
≥94–101.9 cm		9.8	198	45.2	1,369	15.0	149	28.3	1,716
≥102 cm		0.4	8	16.7	507	79.5	792	21.5	1,307
**Physical activity**	0.6								
Inactive		16.1	327	18.3	555	22.6	225	18.4	1,115
Moderately inactive		29.8	603	31.5	954	28.7	286	30.4	1,845
Moderately active		26.1	529	24.6	746	24.7	246	25.1	1,525
Active		26.0	526	24.1	731	22.9	228	24.5	1,490
**Highest school level**	0.7								
None		1.9	38	5.7	174	11.8	118	5.4	330
Primary		26.1	529	35.8	1,083	42.2	420	33.6	2,037
Technical		24.2	490	22.7	686	18.9	188	22.6	1,370
Secondary		15.4	312	12.2	370	9.7	97	12.9	786
Further education		30.6	619	22.1	670	15.6	155	23.8	1,445
**Smoking status**	0.5								
Never		34.3	694	31.0	938	26.4	263	31.3	1,900
Former		32.0	649	37.8	1,146	40.0	398	36.2	2,197
Current		32.3	655	30.1	911	32.5	324	31.3	1,901
**Family history of diabetes** b	8.9								
Yes		11.3	142	16.4	260	17.3	69	14.5	471
No		88.7	1,117	83.6	1,327	82.7	329	85.5	2,773

Data are means and SDs for continuous and percentages and frequencies for categorical variables. Twenty men in the subcohort were underweight (BMI<18.5 kg/m^2^) and were excluded from this table; 41 men in the subcohort had missing values for BMI and were excluded from this table.

a Data on WC and waist-hip ratio were not collected at the centre in Umea, Sweden (excluded from these summaries).

b Family history data were not collected at the centres in Italy, Spain, Oxford, or Heidelberg, so these countries have been excluded when calculating percentages of individuals with/without a family history of diabetes.

**Table 2:** Characteristics of the subcohort by BMI group in women of the InterAct study.

Characteristic	Missing (Percent)	Normal, 18.5–24.9 kg/m^2^ (*n* = 4,942)		Overweight, 25.0–29.9 kg/m^2^ (*n* = 3,322)		Obese, ≥30.0 kg/m^2^ (*n* = 1,558)		Total (*n* = 9,822)	
**Age (years)**	0.0	51.1	9.6	53.3	8.9	53.2	8.4	52.1	9.3
**Weight (kilograms)**	0.4	59.6	6.1	70.0	6.3	84.7	11.0	66.8	11.7
**Height (centimetres)**	0.2	162.9	6.5	160.4	6.4	158.8	6.8	161.4	6.7
**WC (centimetres)** a	3.4	73.8	6.0	84.7	6.5	98.1	9.2	81.2	11.2
**Waist-hip ratio (percent)** a	3.5	77.1	5.5	81.4	5.9	85.0	6.4	79.8	6.5
**Weight at age 20 y (kilograms)**	38.8	55.0	6.7	57.1	7.2	61.3	10.4	56.4	7.7
**Change in weight (kilograms)**	38.8	5.1	6.5	14.1	7.0	26.1	11.5	10.6	10.4
**Duration of follow-up (years)**	0.0								
Non-case		12.1	1.9	12.3	1.9	12.3	2.0	12.2	1.9
Incident diabetes case		8.0	3.2	7.7	3.1	7.0	3.1	7.4	3.1
**WC groups** a	3.4								
<80 cm		78.2	3,863	20.7	688	0.8	13	47.0	4,691
≥80–87.9 cm		13.4	661	45.5	1,513	9.1	141	23.2	2,315
≥88 cm		1.8	87	29.7	988	86.6	1,350	24.3	2,425
**Physical activity**	0.7								
Inactive		20.1	992	29.1	966	42.0	655	26.5	2,648
Moderately inactive		35.4	1,750	35.5	1,179	31.1	485	34.7	3,462
Moderately active		23.7	1,171	18.8	625	14.2	221	20.6	2,059
Active		19.6	969	15.6	518	11.4	177	16.9	1,690
**Highest school level**	1.2								
None		2.4	119	11.3	374	23.5	366	8.6	860
Primary		25.8	1,274	38.6	1,281	39.8	620	32.1	3,204
Technical		24.9	1,231	22.8	757	17.7	276	23.0	2,299
Secondary		20.3	1,003	13.5	449	8.4	131	16.3	1,624
Further education		24.7	1,220	12.2	405	8.3	130	18.0	1,796
**Smoking status**	0.7								
Never		50.0	2,471	58.6	1,948	66.6	1,037	55.4	5,527
Former		22.7	1,122	20.8	690	17.5	272	21.1	2,104
Current		26.2	1,294	19.7	655	14.8	231	22.4	2,238
**Family history of diabetes** b	7.2								
Yes		17.4	531	22.4	357	27.3	158	20.1	1,046
No		82.6	2,513	77.6	1,237	72.7	420	80.0	4,170

Data are means and SDs for continuous and percentages and frequencies for categorical variables. 152 women in the subcohort were underweight (BMI<18.5 kg/m^2^) and were excluded from this table; 69 women in the subcohort had missing values for BMI and were excluded from this table.

a Data on WC and waist-hip ratio were not collected at the centre in Umea, Sweden (excluded from these summaries).

b Family history data were not collected at the centres in Italy, Spain, Oxford, or Heidelberg, so these countries have been excluded when calculating percentages of individuals with/without a family history of diabetes.

### Contributions of BMI and Waist Circumference to the Hazard of T2D

**Figure 1:**
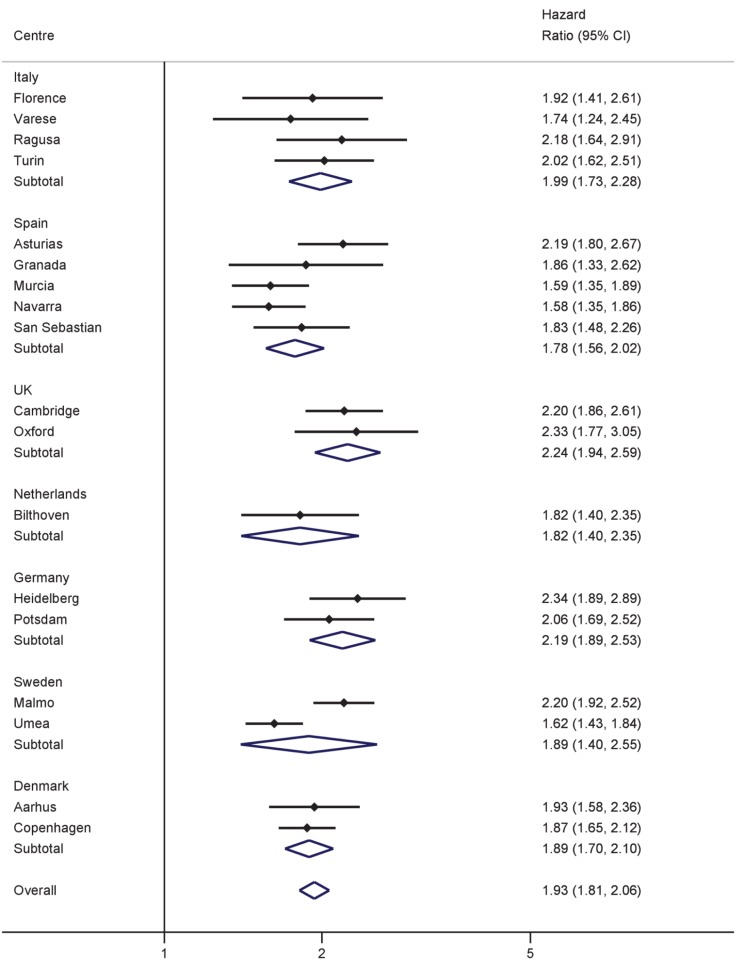
Hazard ratios for type 2 diabetes per 1 SD increase in BMI (SD = 3.6 kg/m^2^) in men. Heterogeneity between centres: *I* ^2^ = 48% (*p* = 0.012). HRs estimated from modified Cox regression with age as the underlying time scale, using Prentice weights. Centre-specific estimates are combined using random effects meta-analysis.

**Figure 2:**
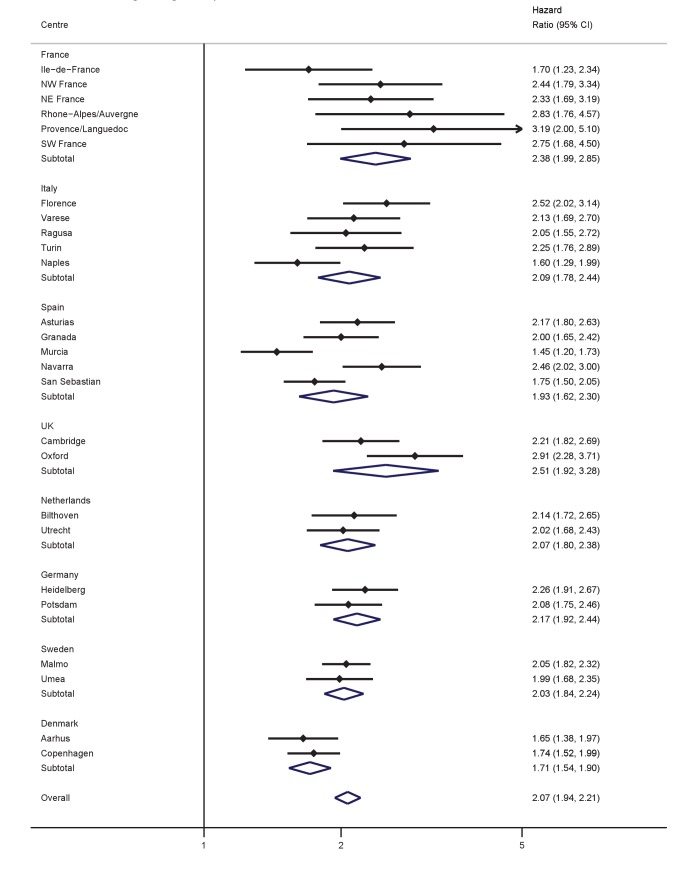
Hazard ratios for type 2 diabetes per 1 SD increase in BMI (SD = 4.4 kg/m^2^) in women. Heterogeneity between centres: *I* ^2^ = 59% (*p* = 0.012). HRs estimated from modified Cox regression with age as the underlying time scale, using Prentice weights. Centre-specific estimates are combined using random effects meta-analysis.

**Figure 3:**
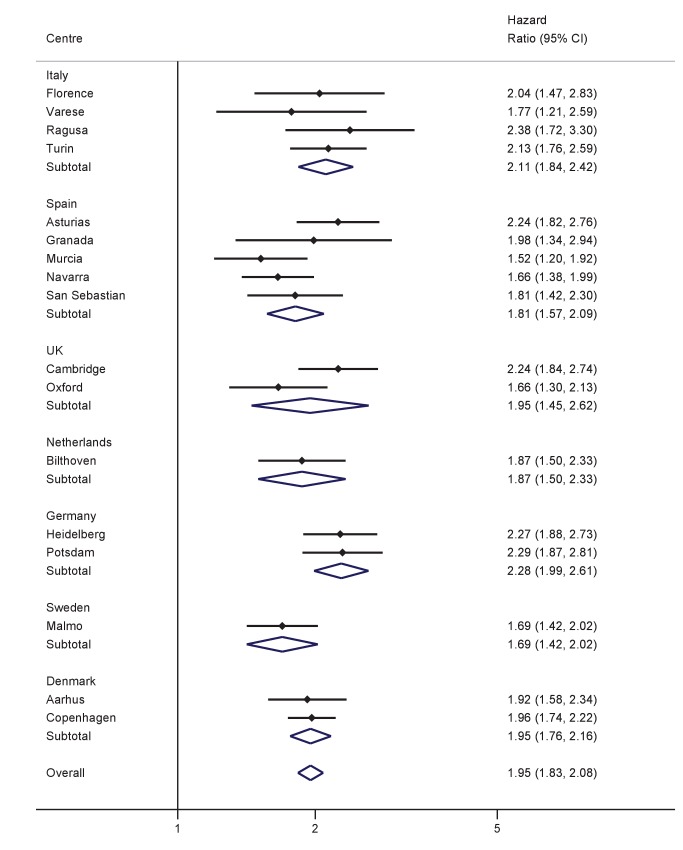
Hazard ratios for type 2 diabetes per 1 SD increase in WC (SD = 10.0 cm) in men. Heterogeneity between centres: *I* ^2^ = 31% (*p* = 0.11). HRs estimated from modified Cox regression with age as the underlying time scale, using Prentice weights. Centre-specific estimates are combined using random effects meta-analysis.

**Figure 4:**
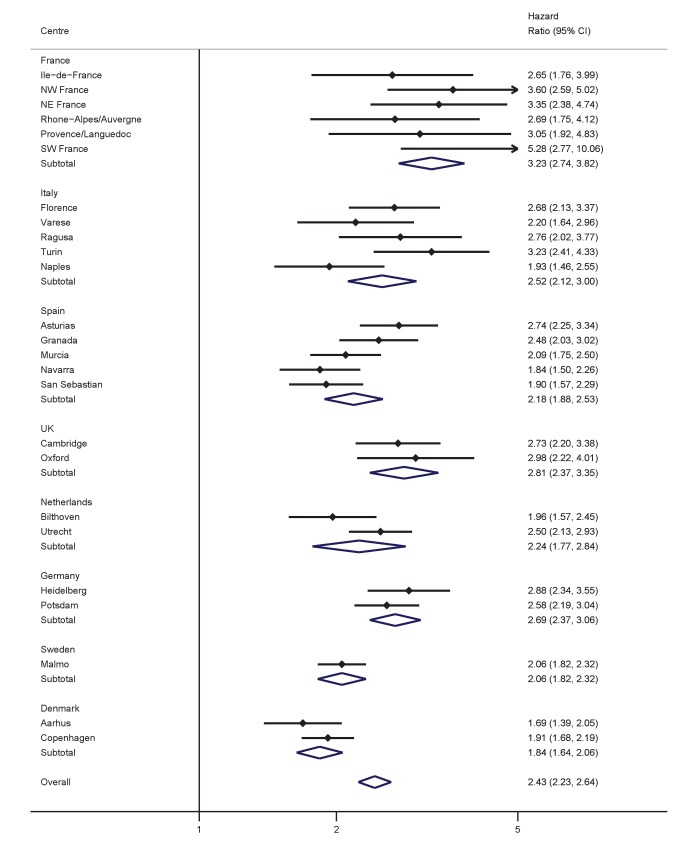
Hazard ratios for type 2 diabetes per 1 SD increase in WC (SD = 11.2 cm) in women. Heterogeneity between centres: *I* ^2^ = 69% (*p*<0.001). HRs estimated from modified Cox regression with age as the underlying time scale, using Prentice weights. Centre-specific estimates are combined using random effects meta-analysis.

Significant, positive associations between both BMI and WC and the hazard of T2D were observed across all countries and centres in men and women (Figures 1–4). The pooled effect estimate (HR) for a 1 SD increase in BMI (SD 3.6 kg/m^2^ in men, 4.4 kg/m^2^ in women) was 1.93 (95% confidence interval 1.81; 2.06) in men and 2.07 (1.94; 2.21) in women; corresponding estimates for WC (SD 10.0 cm in men, 11.2 cm in women) were 1.95 (1.83; 2.08) in men and 2.43 (2.23; 2.64) in women (Figures 1–4; Table 3). There was heterogeneity between centres in the HRs for both BMI and WC (Figures 1–4), which was not explained by differences in the average age of participants in the different centres. A higher average WC was associated with a lower HR per 1 SD increase in BMI; inclusion of average WC in a meta-regression model reduced the *I* ^2^ values from 48% to 0% in men and from 59% to 52% in women. However, average BMI did not explain the heterogeneity in the WC to T2D associations in either men or women. There was no significant interaction between BMI and WC in either men (interaction parameter estimate 0.97 [0.85; 1.11], *p* = 0.66) or women (interaction parameter estimate 1.10 [0.99; 1.22], *p* = 0.073).

**Table 3:** Anthropometric measures and incident type 2 diabetes.

Measure	Men		Women	
	SD	HR (95% CI)	SD	HR (95% CI)
**BMI**				
18.5–24.9 kg/m^2^		1		1
25.0–29.9 kg/m^2^		2.84 (2.33, 3.45)		3.81 (3.18, 4.58)
≥30.0 kg/m^2^		7.58 (6.00, 9.57)		11.6 (9.20, 14.5)
**WC**				
<94/80 cm		1		1
≥94–101.9/80–87.9 cm		2.40 (2.01, 2.88)		3.02 (2.61, 3.48)
≥102/88 cm		5.36 (4.38, 6.54)		11.3 (9.15, 13.9)
**Per 1 SD increase in BMI**	3.6		4.4	
Unadjusted		1.93 (1.81, 2.06)		2.07 (1.94, 2.21)
Adjusted for WC		1.49 (1.27, 1.73)		1.15 (1.02, 1.30)
Adjusted for WC, physical activity, smoking, and education		1.58 (1.36, 1.83)		1.16 (1.01, 1.34)
**Per 1 SD increase in WC**	10.0		11.2	
Unadjusted		1.95 (1.83, 2.08)		2.43 (2.23, 2.64)
Adjusted for height		2.03 (1.88, 2.20)		2.46 (2.27, 2.68)
Adjusted for BMI		1.39 (1.19, 1.63)		2.14 (1.87, 2.45)
Adjusted for BMI, physical activity, smoking, and education		1.37 (1.19, 1.58)		2.25 (1.90, 2.66)
**Per 1 SD increase in waist-hip ratio**	6.3		6.5	
Unadjusted		1.81 (1.61, 2.03)		2.15 (1.93, 2.40)
Adjusted for BMI		1.38 (1.25, 1.52)		1.71 (1.57, 1.86)
Adjusted for BMI, physical activity, smoking, and education		1.33 (1.21, 1.47)		1.75 (1.58, 1.93)

HRs estimated from modified Cox regression with age as the underlying time scale, using Prentice weights. Centre-specific estimates are combined using random effects meta-analysis.

### Differences in Associations between Men and Women

The stronger association between WC and incident T2D in women, compared to men, became more apparent in models mutually adjusting for WC and BMI (Table 3). While the independent contributions of a sex-specific SD increase in WC and BMI to the hazard of diabetes were of similar magnitude in men (1.39 [1.19; 1.63] for WC and 1.49 [1.27; 1.73] for BMI), the increased hazard of diabetes conveyed by a larger WC as opposed to higher BMI was much larger in women (2.14 [1.87; 2.45] for WC and 1.15 [1.02; 1.30] for BMI). A disproportionately increased HR of diabetes in women (HR 11.3 [9.15; 13.9]) compared to men (HR 5.36 [4.38; 6.54]; Table 3) was also seen using sex-specific cut-offs clinically used to identify individuals with central adiposity and excess visceral fat (>102 cm [40 inches] in men and >88 cm [35 inches] in women). Consistently greater HRs were observed in women across all study centres (Figure S1), with sex ratios (HR_women_/HR_men_) ranging from 1.03 to 3.31 and a pooled sex ratio of 1.69 (1.42; 2.02). The higher hazard associated with greater WC in women was not explained by confounding by BMI, since the BMI adjusted pooled sex ratio was 1.60 (1.34; 1.90).

### Hazard Ratio of Diabetes at Different Levels of Waist Circumference and BMI

BMI and WC are highly positively correlated (*r* = 0.85 in men and 0.87 in women in the subcohort). Therefore, too few men and women with a BMI of 18.5–22.4 kg/m^2^ had a WC greater than or equal to 94/80 cm (*n* = 130) and too few men with a BMI of 22.5–24.9 kg/m^2^ had a WC greater than or equal to 102 cm (*n* = 13) to contribute to stratified analyses (Tables 4 and 5). The same was true for participants with a BMI of 30–34.9 kg/m^2^ and a WC lower than 94/80 cm (*n* = 49), or those with a BMI greater or equal to 35 kg/m^2^ and a WC lower than 102/88 cm (*n* = 14).

**Table 4:** Combinations of BMI and waist circumference groups and type 2 diabetes in men.

Weight Category	BMI (kg/m^2^)	WC (centimetres)		
		<94	≥94–101.9	≥102
**Underweight**	<18.5			
		20/3	0/0	0/0
**Normal**	18.5–22.4	**1 (Reference group)**		
		625/109	8/3	0/0
	22.5–24.9	**1.53 (1.20, 1.95)**	**3.00 (1.84, 4.89)**	
		1,412/375	308/120	13/5
**Overweight**	25.0–27.4	**2.76 (1.74, 4.37)**	**4.49 (2.74, 7.36)**	**5.67 (2.84, 11.3)**
		1,203/439	1,328/634	248/137
	27.5–29.9	**3.77 (2.53, 5.61)**	**6.05 (3.87, 9.45)**	**8.91 (5.69, 13.9)**
		233/97	1,335/749	1,044/707
**Obese**	30.0–34.9		**7.48 (4.55, 12.3)**	**13.3 (8.32, 21.1)**
		27/16	314/183	2,111/1,550
	≥35.0			**22.0 (14.3, 33.8)**
		0/0	5/4	557/470

HRs estimated from modified Cox regression with age as the underlying time scale, using Prentice weights. Country-specific estimates are combined using random effects meta-analysis. Sample size and case number stated per cell.

**Table 5:** Combinations of BMI and waist circumference groups and type 2 diabetes in women.

Weight Category	BMI (kg/m^2^)	WC (centimetres)		
		<80	≥80–87.9	≥88
**Underweight**	<18.5			
		139/13	0/0	1/1
**Normal**	18.5–22.4	**1 (Reference group)**		
		2,325/257	108/28	14/4
	22.5–24.9	**1.71 (1.39, 2.09)**	**3.47 (2.70, 4.45)**	**5.55 (3.44, 8.95)**
		2,100/344	798/228	115/40
**Overweight**	25.0–27.4	**2.47 (1.93, 3.18)**	**5.10 (4.16, 6.25)**	**10.3 (8.00, 13.3)**
		765/165	1,430/497	659/338
	27.5–29.9	**4.90 (3.12, 7.69)**	**5.82 (4.64, 7.31)**	**13.7 (10.1, 18.6)**
		120/41	867/333	1,375/778
**Obese**	30.0–34.9		**6.10 (4.29, 8.66)**	**18.6 (14.6, 23.8)**
		22/9	229/94	2,512/1,646
	≥35.0			**31.8 (25.2, 40.2)**
		0/0	9/4	1,271/980

HRs estimated from modified Cox regression with age as the underlying time scale, using Prentice weights. Country-specific estimates are combined using random effects meta-analysis. Sample size and case number stated per cell.

Compared to those with a BMI of 18.5–22.4 kg/m^2^ and normal WC, the HR of diabetes was successively higher at greater levels of BMI and WC, ranging from 1.53 (1.20; 1.95) in men and 1.71 (1.39; 2.09) in women with normal weight at BMI 22.5–24.9 kg/m^2^ and a normal WC, to 22.0 (14.3; 33.8) in men and 31.8 (25.2; 40.2) in women with a BMI greater or equal to 35 kg/m^2^ and a high WC. The HR of diabetes was generally higher (or similar) when comparing people in a lower BMI but higher WC group to those in the BMI group above with a smaller WC, an effect that was particularly pronounced in women. For example, in overweight women, the HR in those with a BMI of 25.0–27.4 kg/m^2^ and a large WC (≥88 cm) was 10.3 (8.00; 13.3), but was 5.82 (4.64; 7.31) in those with a BMI of 27.5–29.9 kg/m^2^ and a moderately increased WC (≥80–87.9 cm).

### Cumulative 10-y Incidence of Developing Diabetes

**Figure 5:**
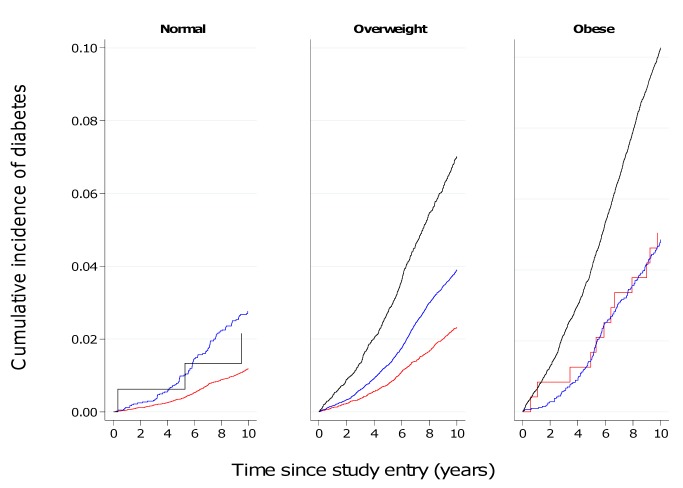
Cumulative incidence of type 2 diabetes over 10 y by BMI and waist circumference groups in men. Red line, WC<94 cm; blue line, WC≥94–101.9 cm; black line, WC≥102 cm.

**Figure 6:**
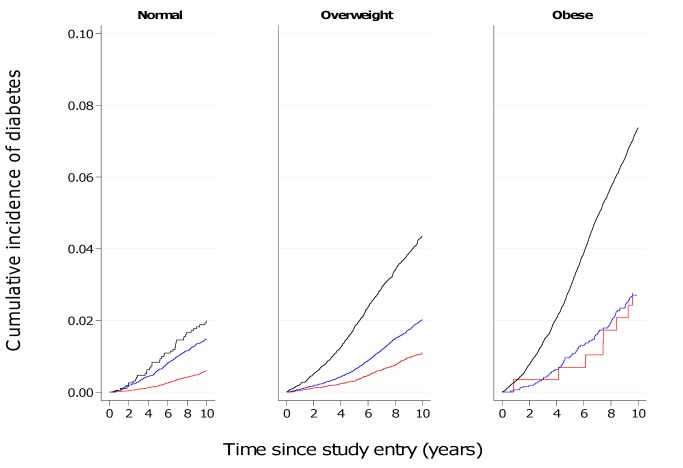
Cumulative incidence of type 2 diabetes over 10 y by BMI and waist circumference groups in women. Red line, WC<80 cm; blue line, WC≥80–87.9 cm; black line, WC≥88 cm.

Figures 5 and 6 show the cumulative incidence of T2D over 10 y of follow-up for different groups of BMI and WC, separately in men and women. Tables S1 and S2 additionally include cumulative incidences for three different follow-up times (5, 10, 15 y) together with 95% confidence intervals, numbers of events, and person-years of follow-up. The cumulative 10-y incidences estimated in normal weight participants (BMI 18.5–24.9 kg/m^2^) were 1.2%, 2.8%, and 2.2% in men with a normal (<94 cm), moderately increased (≥94–101.9 cm), and large (≥102 cm) WC; the corresponding figures for women with a normal (<80 cm), moderately increased (≥80–87.9 cm), and large (≥88 cm) WC were 0.59%, 1.5%, and 2.0%. In overweight individuals, WC distinguished those with incidence rates comparable to normal weight from those with rates equivalent to obese individuals. Cumulative 10–y incidences for men with normal, moderately increased, and large WC were 2.3%, 3.9%, and 7.0% in overweight men and 5.0%, 4.9%, and 10.3% in obese men. Corresponding figures were 1.1%, 2.0%, and 4.4% in overweight women and 2.8%, 2.7%, and 7.4% in obese women, respectively.

## Discussion

Results based on 12,403 incident cases of T2D identified in 26 centres in eight European countries as part of the InterAct case-cohort study show independent, significant contributions of both BMI and WC to the risk of T2D. We found greater HRs for WC in women, compared to men, in analyses of standardised, continuous measures as well as using recommended clinical thresholds for abdominal obesity. In terms of absolute risk, 7% of men and 4.4% of women who were overweight and had a large WC at baseline developed diabetes over a 10-y period, placing them at an absolute risk equivalent to or higher than that of obese participants.

### Clinical Implications

In addition to obese and severely obese individuals at high risk of diabetes, more than a third of the population in the US and UK is overweight [3],[4]. These individuals' risk of T2D is much less well defined, despite its potentially greater contribution to the absolute burden of diabetes and related complications. We show that assessment of WC identifies those at high risk of T2D among the large group of individuals who are overweight. Individual lifestyle interventions can reduce diabetes risk [5], but are not feasible in everyone who is overweight or obese. Current clinical practice relies on BMI to identify overweight and obese individuals at increased risk of diabetes and other adiposity-related morbidity and mortality. Although measurement of WC is often recommended in clinical guidelines, it is rarely actually performed. In a survey of practice nurses in the UK, 96% reported measuring BMI in a typical week, but only 12% measured WC [20]. Recommendations to measure WC on everyone are unlikely to be successful since time pressures are cited as one of the explanations for the implementation gap between recommendations and actual clinical practice. Thus risk prediction models for T2D that assume universal measurement are unlikely to be helpful. As an alternative strategy, practice could focus measurement of WC on subgroups in whom the additional information is likely to make a difference to clinical decision making. Our results suggest that current clinical recommendations should consider the introduction of WC measurement amongst all overweight men and women to identify high-risk individuals for early lifestyle intervention. Normal weight men and women were at sufficiently low absolute risk that measurement of WC would not change their risk categorisation. People in the obese group should already be targeted for individualised lifestyle intervention programmes, and measurement of WC would not alter this recommendation. This observation, of course, does not imply that WC is an unimportant aetiological risk factor in normal weight and obese individuals, but rather that its measurement in these groups does not have an impact on clinical decision making.

### Public Health Implications

The frequency of diabetes in combination with its severe long-term complications through organ damage and dysfunction, particularly of the cardiovascular system, create a major public health problem and serious burden on health care systems. Obesity is the strongest risk factor for T2D, and World Health Organization projections estimate that by 2015 approximately 2.3 billion adults will be overweight, more than 700 million will be obese, and diabetes deaths will increase by more than 50% worldwide during this time [21]. Prevention strategies for T2D require a balance of investment between population-level interventions aimed at shifting the whole distribution of key risk factors and individually focused lifestyle interventions targeted at high-risk individuals. Our results clearly show the value that measurement of WC may have in identifying which people among the large population of overweight individuals are at highest risk of diabetes.

### Comparison with Previous Studies

Previous literature-based reviews have investigated associations between BMI, WC, or other measures of abdominal obesity and incident diabetes [7],[8],[22] and reported similar associations, with risks being approximately twice as large per 1 SD difference in the different obesity measures. Results from the EPIC Potsdam cohort including 1,008 incident T2D cases suggested that the relative risk of T2D associated with WC is smaller in obese than in normal and overweight men and women [23]. We found no evidence for a significant interaction between BMI and WC in men or women in the InterAct study, which includes incident cases from the EPIC Potsdam cohort. Individual studies and meta-analyses investigating the separate and joint contributions of measures of overall and central adiposity have often focused on their respective aetiological relevance [2],[7],[8],[23]. However, it is difficult to draw inference about the specific role of central and abdominal obesity for diabetes development from epidemiological analyses mutually adjusting for BMI and WC given the strong correlation between these measures and differences in their respective measurement errors [9].

Although associations of BMI and WC with T2D incidence did not differ substantially between countries, and effect estimates of BMI and WC appeared largely consistent, we observed significant heterogeneity in our meta-analyses, with *I* ^2^ values of 48% and 31% for BMI and WC in men and 59% and 69% in women. Meta-regression analyses showed that this heterogeneity was not explained by differences in the average age of participants from the different countries. In contrast, centres with higher average WCs tended to have lower effect estimates of BMI, and inclusion of average WC in meta-regression analyses reduced the *I* ^2^ in men from 48% to 0% and in women from 59% to 52%. Average BMI did not explain heterogeneity in the association between WC and T2D in men and women.

### Sex Differences

We observed a stronger effect estimate for excess abdominal fat in women compared to men using different analytical strategies. This was not due to sex differences in the correlation between WC and BMI, which was similar in both sexes (0.86 in men and 0.87 in women in the subcohort). Also, sex differences in the association between WC and T2D were not explained by height, which was shown to only have a very marginal influence on the association. Previous work has highlighted the value of WC as an index of abdominal fat accumulation when it is interpreted in the context of overall levels of adiposity [24],[25]. In this study, sex differences in the association between WC and T2D were particularly pronounced after adjusting for BMI. This suggests that WC may be a better measure of abdominal fat and diabetes risk in women once differences in overall body size are accounted for, potentially because of a greater contribution of subcutaneous fat to women's WC levels, compared to men.

These results demonstrate that women at greater relative risk of T2D are identified when using recommended, sex-specific WC cut-offs and suggest that cut-offs need to be reviewed if the aim is to target comparable levels of relative risk in men and women. However, if the aim is to target groups based on absolute risk, then the observation that absolute levels of T2D risk are lower in women at any level of WC compared to men is more important.

### Strengths and Weaknesses

This is the largest study of incident diabetes to date to investigate the separate and joint contributions of BMI and WC. Advantages of our study include its power, prospective design, and international, multicentre population. Inclusion of over 12,000 incident cases allows investigation of T2D risk for different combinations of BMI and WC cut-offs with greater precision. The prospective design of the InterAct case-cohort study minimises systematic error introduced by recall or treatment bias that cross-sectional and case-control studies are subject to. Investigation on a Europe-wide scale increases the generalisability of our findings. However, while the possibility of examining anthropometric effects across the eight European countries can help to understand factors contributing to any potential heterogeneity, results from our European descent InterAct participants do not allow inferences about BMI- and WC-associated relative or absolute risks of T2D in other ethnic groups with potentially different body composition and T2D incidence. Methods for case ascertainment and verification in InterAct are largely based on a clinical diagnosis of T2D. Estimates of the cumulative incidence in InterAct are therefore an underestimation, potentially differential with regard to obesity levels, as rates are expected to be higher if undiagnosed, asymptomatic diabetes cases were also considered. While our large-scale study had standardised measures of anthropometry available for all except 363 Oxford participants, some differences existed between centres in terms of the WC measurement site or the clothes worn during measurement. Assuming that any misclassification was non-differential with regard to case status, this may have led to an attenuation of the observed associations for WC, highlighting the importance of appropriately designed and powered studies with standardised measures of WC to address its relative importance for the risk of diabetes and other outcomes.

### Conclusion

WC is independently and strongly associated with T2D, particularly in women, and should be more widely measured. If targeted measurement is necessary for reasons of resource scarcity, measuring WC in overweight individuals may be an effective strategy since it identifies a high-risk subgroup of individuals who could benefit from individualised preventive action.

## Supporting Information

Figure S1 **Ratio (women/men) of hazard ratios for the effect of an increased waist circumference (≥102 cm in men and ≥88 cm in women) on incident type 2 diabetes.**
(TIF)

Table S1 **Cumulative incidence of type 2 diabetes by BMI and waist circumference in men.**
(DOC)

Table S2 **Cumulative incidence of type 2 diabetes by BMI and waist circumference in women.**
(DOC)

## Abbreviations

BMI: body mass index
EPIC: European Prospective Investigation into Cancer and Nutrition
HR: hazard ratio
SD: standard deviation
T2D: type 2 diabetes
WC: waist circumference
