# Supplementary figures and images for: Long-Term Risk of Incident Type 2 Diabetes and Measures of Overall and Regional Obesity: The EPIC-InterAct Case-Cohort Study
Source: PLoS Med. 2012 Jun 5;9(6):e1001230. doi: 10.1371/journal.pmed.1001230 (PMC3367997; doi:10.1371/journal.pmed.1001230)

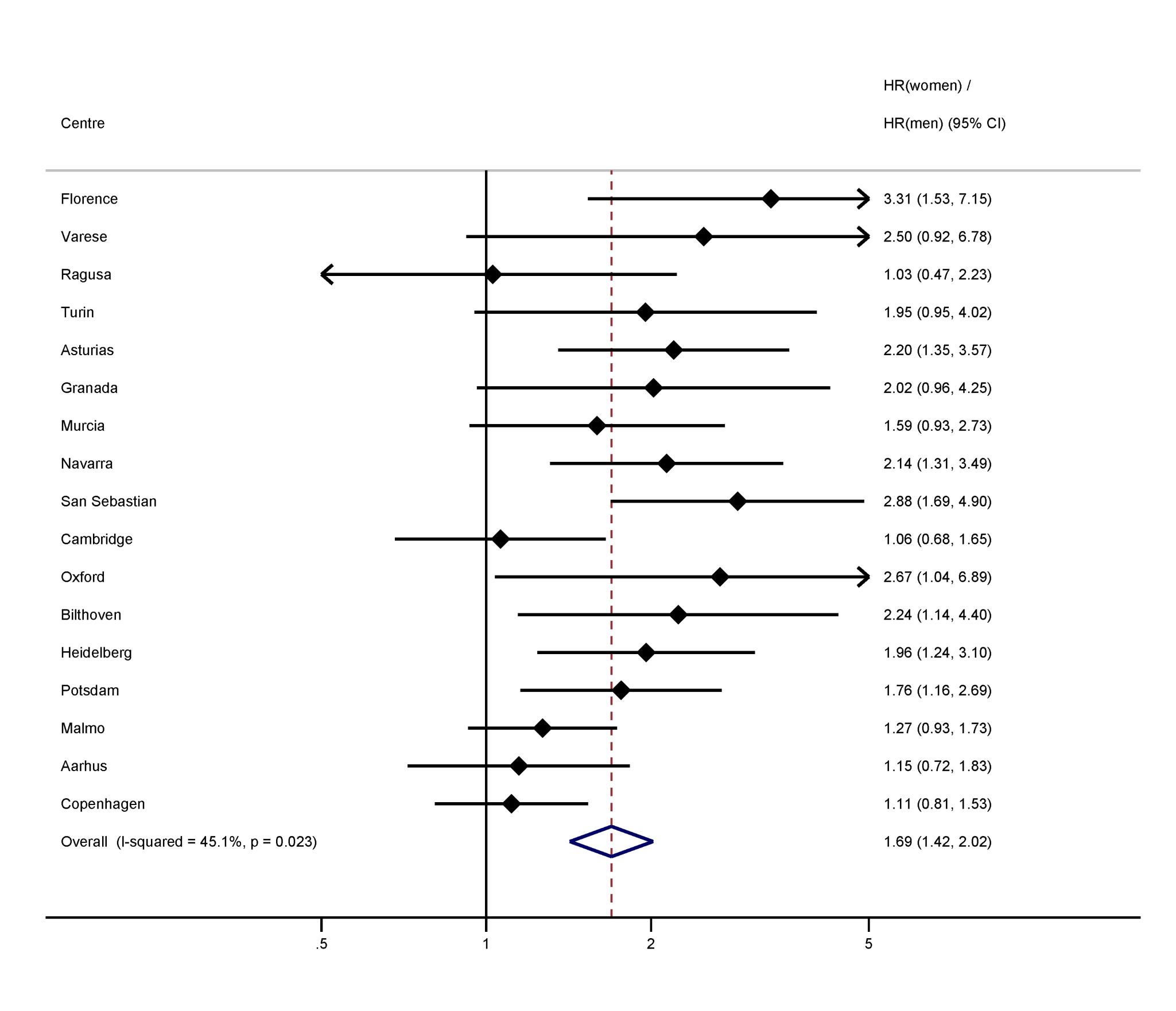

Supplement: Figure S1 — Ratio (women/men) of hazard ratios for the effect of an increased waist circumference (≥102 cm in men and ≥88 cm in women) on incident type 2 diabetes. (TIF) [file pmed.1001230.s001.tif]
