# Supplementary material for: Long-Term Risk of Incident Type 2 Diabetes and Measures of Overall and Regional Obesity: The EPIC-InterAct Case-Cohort Study
Source: PLoS Med. 2012 Jun 5;9(6):e1001230. doi: 10.1371/journal.pmed.1001230 (PMC3367997; doi:10.1371/journal.pmed.1001230)
Supplement: Table S1 — Cumulative incidence of type 2 diabetes by BMI and waist circumference in men. (DOC) [file pmed.1001230.s002.doc]

**Table S1.** Cumulative incidence of T2D by BMI and waist circumference in men.

| **Men** |  |  |  |  |  |
| --- | --- | --- | --- | --- | --- |
|  | **BMI (kg/m^2^)** | **Waist (cm)** | **Follow-up time** | **N (events)/ PY** | **Cumulative Incidence (%)**  **(95% CI)** |
|  |  |  |  |  |  |
| **Normal** | 18.5-24.9 | <94 | 5 years | 111/158034 | 0.35 (0.29,0.42) |
|  |  |  | 10 years | 366/310007 | 1.2 (1.1,1.3) |
|  |  |  | 15 years | 484/381518 | 2.0 (1.8,2.2) |
|  |  |  |  |  |  |
|  |  | >94-101.9 | 5 years | 39/19916 | 0.99 (0.72,1.4) |
|  |  |  | 10 years | 104/37789 | 2.8 (2.3,3.3) |
|  |  |  | 15 years | 123/46270 | 4.0 (3.3,4.9) |
|  |  |  |  |  |  |
|  |  | ≥102 | 5 years | 1/782 | 0.62 (0.09,4.3) |
|  |  |  | 10 years | 3/1403 | 2.2 (0.69,6.6) |
|  |  |  | 15 years | 5/1590 | - |
|  |  |  |  |  |  |
| **Overweight** | 25.0-29.9 | <94 | 5 years | 143/94422 | 0.76 (0.64,0.89) |
|  |  |  | 10 years | 431/185078 | 2.3 (2.1,2.6) |
|  |  |  | 15 years | 536/230109 | 3.3 (3.0,3.6) |
|  |  |  |  |  |  |
|  |  | >94-101.9 | 5 years | 361/140985 | 1.3 (1.2,1.4) |
|  |  |  | 10 years | 1068/272005 | 3.9 (3.7,4.1) |
|  |  |  | 15 years | 1383/336270 | 5.9 (5.6,6.3) |
|  |  |  |  |  |  |
|  |  | ≥102 | 5 years | 289/52457 | 2.7 (2.4,3.1) |
|  |  |  | 10 years | 702/98244 | 7.0 (6.5,7.5) |
|  |  |  | 15 years | 844/119920 | 9.6 (8.9,10.3) |
|  |  |  |  |  |  |
| **Obese** | ≥30.0 | <94 | 5 years | 4/1180 | 1.7 (0.63,4.4) |
|  |  |  | 10 years | 12/2330 | 5.0 (2.9,8.7) |
|  |  |  | 15 years | 16/2894 | - |
|  |  |  |  |  |  |
|  |  | >94-101.9 | 5 years | 46/15531 | 1.5 (1.1,2.0) |
|  |  |  | 10 years | 143/29412 | 4.9 (4.1,5.7) |
|  |  |  | 15 years | 187/37188 | 7.0 (6.0,8.0) |
|  |  |  |  |  |  |
|  |  | ≥102 | 5 years | 689/84393 | 4.0 (3.7,4.3) |
|  |  |  | 10 years | 1638/154872 | 10.3 (9.8,10.7) |
|  |  |  | 15 years | 2020/186263 | 14.8 (14.1,15.6) |

*Note: The cumulative incidence is a probability (expressed as a percentage) of experiencing diabetes over 5, 10 or 15 years of follow-up. N/PY is the observed rate of diabetes. These do not therefore represent the same quantity. N/PY and cumulative incidences are estimated by resampling with replacement from the subcohort.*
