# Supplementary material for: Long-Term Risk of Incident Type 2 Diabetes and Measures of Overall and Regional Obesity: The EPIC-InterAct Case-Cohort Study
Source: PLoS Med. 2012 Jun 5;9(6):e1001230. doi: 10.1371/journal.pmed.1001230 (PMC3367997; doi:10.1371/journal.pmed.1001230)
Supplement: Table S2 — Cumulative incidence of type 2 diabetes by BMI and waist circumference in women. (DOC) [file pmed.1001230.s003.doc]

**Table S2.** Cumulative incidence of T2D by BMI and waist circumference in women.

| **Women** |  |  |  |  |  |
| --- | --- | --- | --- | --- | --- |
|  | **BMI (kg/m^2^)** | **Waist (cm)** | **Follow-up time** | **N (events)/ PY** | **Cumulative Incidence (%)**  **(95% CI)** |
|  |  |  |  |  |  |
| **Normal** | 18.5-24.9 | <80 | 5 years | 142/416438 | 0.17 (0.14,0.20) |
|  |  |  | 10 years | 485/820339 | 0.59 (0.54,0.65) |
|  |  |  | 15 years | 601/997548 | 0.91 (0.82,1.0) |
|  |  |  |  |  |  |
|  |  | >80-87.9 | 5 years | 88/71890 | 0.61 (0.50,0.75) |
|  |  |  | 10 years | 211/141062 | 1.5 (1.3,1.7) |
|  |  |  | 15 years | 256/171283 | 2.2 (1.8,2.7) |
|  |  |  |  |  |  |
|  |  | ≥88 | 5 years | 16/9587 | 0.83 (5.1,13.5) |
|  |  |  | 10 years | 38/18897 | 2.0 (1.5,2.7) |
|  |  |  | 15 years | 44/22857 | 2.5 (1.8,3.3) |
|  |  |  |  |  |  |
| **Overweight** | 25.0-29.9 | <80 | 5 years | 46/74651 | 0.31 (0.23,0.41) |
|  |  |  | 10 years | 162/147141 | 1.1 (0.95,1.3) |
|  |  |  | 15 years | 206/184581 | 1.7 (1.4,1.9) |
|  |  |  |  |  |  |
|  |  | >80-87.9 | 5 years | 205/165467 | 0.62 (0.54,0.71) |
|  |  |  | 10 years | 656/324187 | 2.0 (1.9,2.2) |
|  |  |  | 15 years | 830/402346 | 3.1 (2.8,3.4) |
|  |  |  |  |  |  |
|  |  | ≥88 | 5 years | 395/109432 | 1.8 (1.6,2.0) |
|  |  |  | 10 years | 933/211019 | 4.4 (4.1,4.6) |
|  |  |  | 15 years | 1116/258186 | 6.2 (5.6,6.7) |
|  |  |  |  |  |  |
| **Obese** | ≥30.0 | <80 | 5 years | 2/1440 | 0.69 (0.17,2.7) |
|  |  |  | 10 years | 8/2863 | 2.8 (1.4,5.5) |
|  |  |  | 15 years | 9/3563 | - |
|  |  |  |  |  |  |
|  |  | >80-87.9 | 5 years | 31/15651 | 0.99 (0.70,1.4) |
|  |  |  | 10 years | 84/30794 | 2.7 (2.2,3.3) |
|  |  |  | 15 years | 98/38943 | 3.3 (2.7,4.0) |
|  |  |  |  |  |  |
|  |  | ≥88 | 5 years | 895/152755 | 2.9 (2.7,3.1) |
|  |  |  | 10 years | 2165/287206 | 7.4 (7.1,7.7) |
|  |  |  | 15 years | 2626/351471 | 10.2 (9.8,10.6) |

*Note: The cumulative incidence is a probability (expressed as a percentage) of experiencing diabetes over 5, 10 or 15 years of follow-up. N/PY is the observed rate of diabetes. These do not therefore represent the same quantity. N/PY and cumulative incidences are estimated by resampling with replacement from the subcohort.*
